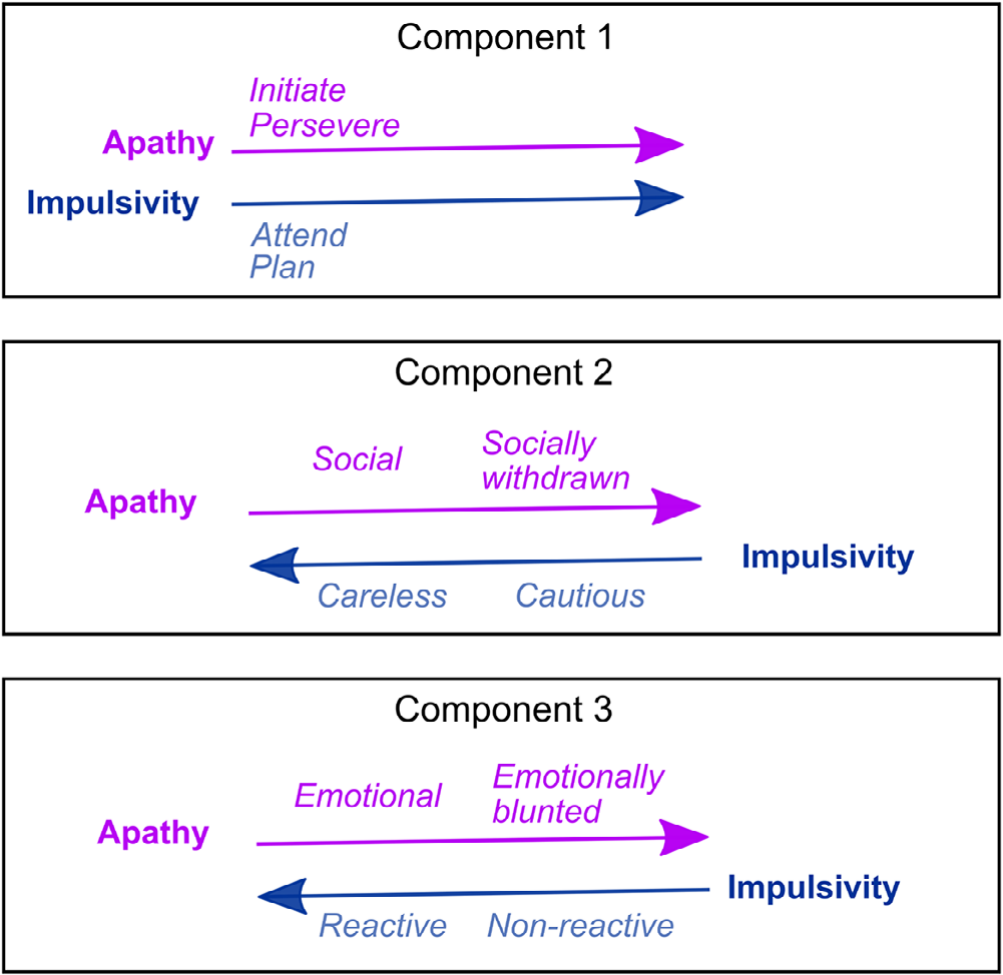# Goal‐directedness deficit in Huntington's disease

**DOI:** 10.1002/alz70857_105286

**Published:** 2025-12-25

**Authors:** Lee‐Anne Morris, Sanjay Manohar, Kyla‐Louise Horne, Laura Paermentier, Christina M Buchanan, Michael MacAskill, Daniel J Myall, Masud Husain, Richard Roxburgh, Tim J Anderson, Campbell J Le Heron

**Affiliations:** ^1^ University of Otago, Christchurch, Canterbury, New Zealand; ^2^ University of Oxford, Oxford, United Kingdom; ^3^ University of Otago, Christchurch, New Zealand; ^4^ New Zealand Brain Research Institute, Christchurch, New Zealand; ^5^ University of Auckland, Auckland, New Zealand

## Abstract

**Background:**

Apathy and impulsivity co‐occur in Huntington's disease (HD), but these debilitating behavioural syndromes are multidimensional constructs, raising the question of which specific dimensions drive this relationship, and the stability of the co‐occurring dimensions across time.

**Methods:**

People with HD and controls completed multidimensional apathy and impulsivity scales at baseline and one‐year follow‐up. A principal component analysis was performed on pooled data (*n* = 109) to identify components and factor loadings of subscales. Linear mixed models were used to examine differences in components between groups and timepoints.

**Results:**

Three meaningful components emerged. Component one comprised positive loading for dimensions of apathy and impulsivity pertaining to goal‐directedness, namely attention, planning, initiation and perseverance. In contrast, other dimensions of apathy and impulsivity loaded onto components two and three in opposite directions. People with HD only scored worse than controls on the goal‐directedness component. All components remained stable over time and each showed remarkable similarity to a factor from the five‐factor personality model, specifically conscientiousness (component one), extraversion (component two) and neuroticism (component three).

**Conclusions:**

The clinical overlap between apathy and impulsivity in HD relates to goal‐directedness, whilst other dimensions of these constructs do not overlap.